# Immunological Criteria for Predicting Severe and Complicated Forms of Chickenpox

**DOI:** 10.17691/stm2020.12.4.06

**Published:** 2020-08-27

**Authors:** O.A. Saburova, T.Yu. Butina, A.M. Ryumin, E.A. Mikhailova, D.M. Sobchak

**Affiliations:** PhD Student, Department of Infectious Diseases; Privolzhsky Research Medical University, 10/1 Minin and Pozharsky Square, Nizhny Novgorod, 603005, Russia; Physician; Academician I.N. Blokhina Nizhny Novgorod Scientific Research Institute of Epidemiology and Microbiology of Rospotrebnadzor (Russian Federal Consumer Rights Protection and Human Health Control Service), 71 Malaya Yamskaya St., Nizhny Novgorod, 603950, Russia; Associate Professor, Department of Infectious Diseases; Privolzhsky Research Medical University, 10/1 Minin and Pozharsky Square, Nizhny Novgorod, 603005, Russia; Associate Professor, Department of Infectious Diseases; Privolzhsky Research Medical University, 10/1 Minin and Pozharsky Square, Nizhny Novgorod, 603005, Russia; Professor, Department of Infectious Diseases Privolzhsky Research Medical University, 10/1 Minin and Pozharsky Square, Nizhny Novgorod, 603005, Russia

**Keywords:** *Varicella zoster*, pro-inflammatory mediators, interferons, cytokines, polymerase chain reaction, neutrophils chemiluminescence, cellular immunity.

## Abstract

**The aim of the study** was to evaluate the levels of mediators of the immune response, cellular immunity, and phagocytic activity in patients with chickenpox with various values of the clinical and laboratory parameters and propose criteria for predicting the severity and complications of the disease.

**Materials and Methods.:**

The blood levels of pro-inflammatory mediators were evaluated by ELISA using monoclonal antibodies (Protein Contour, Russia).

**Results.:**

The inflammatory mediators and neutrophil chemiluminescence were studied in patients with either presence or absence of *Varicella zoster* DNA. We found that in patients with positive viral DNA, the levels of IFN-α and IFN-γ were significantly lower compared to patients with negative DNA results. Thus, complications of chickenpox, in particular secondary viral-bacterial pneumonia, can be predicted based on low (less than double-normal) levels of IL-6 and IFN-γ, induced chemiluminescence, CD16, and CD20. This type of immune response indicates the state of immune deficiency with prevailing suppression of the T-effector and phagocytic mechanisms in these patients.

**Conclusion.:**

Prognosis of the development of severe and complicated forms of chickenpox can be based on the insufficiently increased (less than two normal values) levels of IL-6 and IFN-γ, induced chemiluminescence, CD16, and CD20. These relatively low levels are indicative of reduced immune response to the infection, which may require additional immune correcting therapy.

## Introduction

Herpes viruses, a large family of DNA-containing viruses, are widespread in the human population. They are capable of infecting almost all organs and systems, causing a variety of infectious diseases [1–3].

Both chickenpox and herpes zoster (these diseases share common biological properties) are caused by *Varicella zoster* from the *Herpesviridae* virus family.

These virions are shaped as large particles (150–250 nm in diameter) with their genome — a double-helix linear DNA molecule — encapsulated in a lipid envelope. The virus reproduces in nuclei of infected cells; it is unstable in the environment: with droplets of mucus or saliva, its viability lasts no more than 10–15 min. Ultraviolet radiation, heat, sun rays inactivate the virus in a short time [3–5].

Varicella (chickenpox) refers to anthroponoses: humans are the only reservoir of the infection. The virus is transmitted from the infected individual in the period from the last day of incubation until the 5^th^ day of rash manifestation. The mechanism of contraction is aspirational and the transmission is mainly airborne; transmission by contact is rare. Intrauterine transmission of varicella from a pregnant woman to the fetus is possible.

The site of entry for the virus is the mucous membranes of the respiratory tract. The virus settles in the epithelial cells of the skin and mucous membranes of the respiratory tract and oropharynx. Damage to the intervertebral ganglia, cerebellar cortex and cerebral hemispheres is also described. In the generalized form of the disease, the liver, lungs, and gastrointestinal tract are involved.

Post-infectious immunity is well expressed due to the lifelong persistence of the virus. In patients with reduced immunity, the virus may reactivate and cause recurrent herpes zoster manifestations. The incidence of herpes zoster increases after the age of 60 in people with immunodeficiency, exposure to adverse factors, or exacerbation of concomitant chronic diseases.

According to the clinical classification, typical and atypical forms of chickenpox can be distinguished. The atypical ones include rudimentary, bullous, hemorrhagic, gangrenous, and generalized (visceral) forms.

The incubation period of the disease lasts 11–21 days, usually with no prodromal signs. Some patients may develop a short-term prodromal rash (scarlet fever- or measles-like). Vesicles appear almost simultaneously (or few hours later) with an increase in body temperature. The temperature can rise to 39°, intoxication is increasing. Rashes come in a wavelike manner during 2–4 days; every such wave is accompanied by fever. The rash can be localized on the face, scalp, trunk, extremities, palms, and feet. At first, the rashes are papulous in nature; then, they turn into vesicles within a few hours. The latter dry out after 3 to 4 days followed by the crust formation; the crusts fall off after 2 to 3 weeks. Rashes can be detected on the mucous membrane of the oral cavity, larynx, conjunctiva, genitals. The vesicles on the mucous membranes turn to erosions with yellowish bottom, which then form some epithelium-like layer. The rash is often accompanied by enlarging systemic lymph nodes [2–4].

The most common complications of chickenpox include skin infections and viral-bacterial pneumonia. Of the specific complications, lesions of the central nervous system are notable: namely, encephalitis, meningoencephalitis, serous meningitis.

Herpes viruses are intracellular pathogens that are reproduced in the nuclei of infected cells. All non-tumor viruses rather rapidly cause the death of the infected cells. In contrast, tumor viruses are known for their prolonged interaction with the host cell; the virus modifies the biological properties of the cell and makes it grow faster. Herpes viruses can be adsorbed by lipoproteins of cell receptors. The viral protein is synthesized and then, together with viral DNA, gives rise to new viral particles. The newly-formed nucleocapsid is gradually “pushed out” of the cell nucleus and the cytoplasm. The viral envelope is formed from the nuclear and cytoplasmic components of the host cells; as a result, the cell appearance changes. Clusters of viral particles accumulate in various cell compartments and can be detected by light microscopy [4, 5].

The virion (viral particle) incorporates more than 30 glycoproteins, seven of which are located on the surface; in the human body, they induce the formation of virus-neutralizing antibodies. The viral DNA contains 80 genes (the main ones are *а*, *b*, and *g*). The *a-*genes play a role in the latency phase and reactivation of the viruses; they also activate the expression of *b-*genes. The latter produce DNA polymerase, which is needed for viral DNA formation. Other *b-*proteins activate the expression of host cell genes and initiate the activity of *g-*genes. Membrane glycoproteins promote the viral penetration into the cell and play an important role in herpes immunogenesis [4–6].

In the pathogenesis of chickenpox, T lymphocytes play a significant role by activating cellular immune response. Patients with immunodeficiency develop severe and generalized forms of the disease. After attenuation of the primary infection, the virus can remain in the body for a long time [2–4, 6].

T lymphocytes produce mediators of the immune response and thus activate the cellular and humoral immunity. The key mediator of the immune response is interleukin-1 (IL-1) [4, 5]. It is produced by macrophages and monocytes, Langerhans cells, Kuepfer cells, endothelial cells, keratinocytes, fibroblasts, microglia, natural killers, T lymphocytes, and neutrophils. The main inducers of IL-1 production are peptidoglycans, lipopolysaccharides, and endotoxins. There are more inducers such as cytokines, immune complexes, autolysis products, TNF-α, IFN-α, IFN-β, and IL-1β itself [7, 8]. This interleukin activates the production of cytokines and interferon as well as the growth and differentiation of T and B lymphocytes and natural killers. It promotes the development of inflammation, activates the nervous and endocrine system, stimulates bone marrow hematopoiesis, and regeneration of damaged tissues. Together with IFN-α, it enhances antiviral protection at the cellular level, increases the synthesis of IL-6 and interferons [9, 10].

IL-6 is an important mediator of the acute phase of inflammation; it stimulates the proliferation and differentiation of B and T cells and leukopoiesis. It is secreted by macrophages, fibroblasts, vascular endothelial cells, T cells, glial, epithelial cells, and skin keratinocytes.

Interferons induce the synthesis of cellular proteins, which block viral replication. They operate in several ways. In response to a stimulus, cells produce a large amount of protein kinase R. This enzyme phosphorylates the translation initiation factor eIF-2 and activates the synthesis of ribonuclease L, which breaks down cellular RNA and inhibits the protein synthesis. Therefore, the interferon-dependent inhibition of translation eIF-2 is deleterious for both the virus and the host cell. In addition, interferons can activate hundreds of genes that play a role in protecting cells from viruses. They are also able to control the virus propagation by activating protein p53, which stimulates cell apoptosis [10, 11].

Interferon activates the synthesis of HLA-I and HLA-II and thus facilitates the exposure of viral antigens to cytotoxic T lymphocytes, natural killer cells, and T-helper cells that synthesize mediators of the immune response. The synthesis of proinflammatory mediators and phagocytosis characterize the interaction between the pathogen and the human body. In this respect, by determining the parameters of immune response in patients with varicella one can predict the course of the disease and its possible complications [9–11].

**The aim of the study** was to evaluate the levels of mediators of the immune response, cellular immunity, and phagocytic activity in patients with chickenpox with various values of the clinical and laboratory parameters and propose criteria for predicting the severity and complications of the disease.

## Materials and Methods

The presence of pro-inflammatory cytokines was evaluated in 88 children with chickenpox and 60 healthy children aged 5 to 12 years. Severe forms of the disease were diagnosed in 20 patients, and moderate to severe forms — in 68 patients. The study was conducted in accordance with the Helsinki Declaration (2013) and approved by the Ethics Committee of the Privolzhsky Research Medical University. Informed consent was obtained from the parents of the patients.

The diagnosis of chickenpox was based on clinical signs and the anamnesis (fever, vesicular rash on the head, torso, and limbs, as well as systemic lymph node enlargement); the *Varicella zoster* DNA test was also run.

In 18 children, the disease was complicated by viral-bacterial pneumonia. The diagnosis of pneumonia was made on the basis of clinical, laboratory, and radiological data (cough, shortness of breath, wet rales, dull percussion sound, positive inflammatory blood test, infiltrative changes in the lungs).

Determination of *Varicella zoster* DNA by PCR was carried out at the Academician I.N. Blokhina Nizhny Novgorod Scientific Research Institute of Epidemiology and Microbiology of Rospotrebnadzor.

The levels of pro-inflammatory mediators in the blood were measured using ELISA and monoclonal antibodies (Protein contour, Russia). In healthy subjects (control), the concentrations of IL-1β, IL-6, IFN-α, and IFN-γ were 59.2±5.3, 53.4±4.5, 48.9±3.2, and 58.5±3.6 pg/ml, respectively.

In patients with varicella, phagocytic activity was assessed using luminol-induced luminescence as modified by Allen (1993) and Mayansky (1996). Both spontaneous chemiluminescence (SCL) and induced chemiluminescence (ICL) were determined with the help of a Beta-2 liquid-scintillation counter (Medapparatura, Russia). In healthy controls, the indicators of SCL and ICL were (45.4±3.3)**·**10^3^ and (215.7±11.4)**·**10^3^ pulse/min.

Subpopulations of immune cells CD4, CD8, CD16, and CD20 were quantified using the specific monoclonal antibodies and the immunofluorescence assay. In control subjects, the respective values were (1.083±0.014)**·**109/L, (1.032±0.016)**·**109/L, (0.516±0.034)**·**109/L, and (0.422± ±0.023)**·**109/L.

**Statistical processing.** We used the Statistica 6.0 software package and the Student’s t-test. The arithmetic mean (M) and the error of the arithmetic mean (m) were calculated. Differences were considered significant at p<0.05.

## Results

Proinflammatory mediators and neutrophil chemiluminescence were measured in patients with various values of the clinical and laboratory parameters.

The indices of immune response were determined in patients with moderate to severe forms of chickenpox. We found higher levels of INF-γ in patients with a moderate course of the disease compared to the severe form; SCL, ICL, IL-1β, IL-6, IFN-α did not significantly differ (Table 1).

**Table 1:** Pro-inflammatory cytokines and neutrophil chemiluminescence indices in patients with chickenpox of various severity and complications (M±m)

Form of the disease	Number of patients	IL-1β (pg/ml)	IL-6 (pg/ml)	IFN-α (pg/ml)	IFN-γ (pg/ml)	SCL, ×10^3^ (pulse/min)	ICL, ×10^3^ (pulse/min)
Moderate course	68	120.2±11.6	126.2±10.4	126.3±3.2	142.5±10.5	77.5±4.2	265.7±10.4
Severe course	20	102.6±10.4	115.4±9.3	118.3±9.4	72.4±6.6	73.7±6.3	245.7±11.2
		р=0.108	р=0.304	р=0.116	р=0.013*	р=0.208	р=0.314
Chickenpox complicated by pneumonia	18	73.8±4.3	62.7±3.2	103.8±6.5	67.4±5.2	54.2±3.4	205.7±9.5
Uncomplicated chickenpox	70	106.4±9.7	137.2±7.5	114.4±8.2	150.2±11.4	62.2±4.6	428.7±10.8
		р=0.174	р=0.025*	р=0.205	р=0.016*	р=0.202	р=0.014*
Control group (healthy)	60	59.2±4.3	53.4±4.5	48.9±3.2	58.5±3.6	45.4±3.3	215.7±11.4

* Statistically significant differences with the control values.

At the next phase of the study, we looked into the immune parameters in patients with complicated forms of chickenpox. The results showed significantly lower levels of IL-6, IFN-γ, and ICL in patients with chickenpox complicated by pneumonia as compared with uncomplicated forms of the disease (see Table 1).

The levels of inflammatory mediators and neutrophil chemiluminescence were then studied in patients with different contents of *Varicella zoster* DNA. Thus, in the presence of viral DNA, the levels of IFN-α and IFN-γ were significantly lower than those in patients with negative *Varicella zoster* DNA results. The SCL and ICL did not significantly differ between the groups (Table 2).

**Table 2:** Levels of proinflammatory cytokines and neutrophil chemiluminescence in varicella patients with various laboratory results (M±m)

Parameters	Number of patients	IL-1β (pg/ml)	IL-6 (pg/ml)	IFN-α (pg/ml)	IFN-γ (pg/ml)	SCL, ×10^3^ (pulse/min)	ICL, ×10^3^ (pulse/min)
*Varicella zoster* DNA positive	30	115.4±10.3	101.3±9.2	68.2±4.7	72.4±6.2	63.5±5.2	235.7±11.3
*Varicella zoster* DNA negative	58	123.5±9.2	108.2±10.5	135.8±10.9	156.3±12.6	72.7±6.8	265.7±10.5
		p=0.163	p=0.214	p=0.003*	p=0.015*	р=0.304	р=0.216
Inflammatory blood reaction	25	138.6±11.4	146.4±10.5	129.6±11.2	114.5±9.6	75.2±5.3	415.7±12.5
No inflammatory blood reaction	63	63.4±4.5	62.5±4.6	112.8±8.4	104.2±8.2	66.2±4.6	228.7±15.4
		p=0.012*	p=0.015*	p=0.102	p=0.118	р=0.202	р=0.002*
Control group	60	59.2±4.3	53.4±4.5	48.9±3.2	58.5±3.6	45.4±3.3	215.7±11.4

* Statistically significant differences with the control values.

The immune response mediators were determined in patients showing either presence or absence of *Varicella zoster* DNA. Thus, IFN-α and IFN-γ were found at significantly higher quantities in patients with negative rather than positive results of *Varicella zoster* DNA tests. The content of IL-1β and IL-6 did not significantly differ between these two groups of patients (see Table 2).

The immune parameters were also determined in patients with various degrees of leukocytosis. It was found that the levels of IL-1β, IL-6, and ICL were significantly higher in patients with high leukocyte counts and signs of inflammation compared with patients who had no leukocytosis. The indices of IFN-α, IFN-γ, and SCL did not markedly change (see Table 2).

The numbers of CD4, CD8, CD16, and CD20 positive cells were determined in blood samples of patients with either presence or absence of *Varicella zoster* DNA. A significant decrease in the level of CD8 positive cells was found in patients with positive rather than negative tests for the viral DNA. The numbers of CD4, CD16, and CD20 cells differed insignificantly (Table 3).

**Table 3:** Indices of cellular immunity in patients with positive and negative *Varicella zoster* DNA, ×10^9^/L (M±m)

Replication of *Varicella* activity *zoster*	of Number patients	CD4	CD8	CD16	CD20
Lack of DNA	58	0.958±0.042	0.835±0.024	0.613±0.035	0.428±0.016
Identification of DNA	30	0.741±0.023	0.416±0.035	0.305±0.027	0.375±0.032
		р=0.118	р=0.015*	р=0.126	p=0.214
Control group (healthy)	60	1.08±0.01	1.03±0.02	0.52±0.03	0.42±0.02

* Statistically significant differences with the control values.

We also compared the counts of CD4, CD8, CD16, and CD20 cells between patients with complicated and uncomplicated forms of the disease. Those with chickenpox complicated by pneumonia had lower levels of CD16 and CD20 than those with uncomplicated forms of the disease. Indicators of CD4 and CD8 changed insignificantly (Table 4).

**Table 4:** Indices of cellular immunity in patients with a complicated form of chickenpox, ×10^9^/L (M±m)

Disease	Number of patients	CD4	CD8	CD16	CD20
Chickenpox complicated by pneumonia	18	0.612±0.032	0.725±0.024	0.403±0.025	0.413±0.032
Uncomplicated chickenpox	70	0.556±0.021	0.509±0.013	0.117±0.016	0.205±0.051
		р=0.225	р=0.218	p=0.025*	p=0.021*
Control group (healthy)	60	1.08±0.01	1.03±0.02	0.52±0.03	0.42±0.02

* Statistically significant differences with the control values.

## Discussion

In the present study, the immune indices were evaluated in patients with moderate-to-severe and severe forms of chickenpox. Significantly higher levels of IFN-γ were found in patients with the moderate compared to the severe course of the disease. The result suggests that a sub-optimal increase in the level of IFN-γ (less than double-normal values) is indicative of an insufficient immune response and predictive of a severe course of the disease.

We also studied the immune parameters in patients with complicated forms of chickenpox. There were mildly increased levels of IL-6, IFN-γ, and ICL (less than double-normal values) in patients with chickenpox complicated by pneumonia. In this group of children, the levels of CD16 and CD20 antigens were lower than those in patients with uncomplicated forms of the disease. Therefore, such insufficient levels of IL-6, IFN-γ, ICL, CD16, and CD20 can be considered as predictive criteria of secondary pneumonia in pediatric patients with chickenpox. The relatively low values of these indices report of immune deficiency in these patients that may be due to suppression of the T-effector and phagocytic mechanisms.

The levels of inflammatory mediators, neutrophil chemiluminescence, and cellular immunity indices were studied in patients with either presence or absence of *Varicella zoster* DNA. It was found that in patients with positive viral DNA results and a low leukocyte count, the levels of IL-1β, IL-6, ICL, IFN-α, IFN-γ, and CD8 were significantly lower compared with children with negative viral DNA tests and persistent leukocytosis. Therefore, an insufficient increase in pro-inflammatory mediators (less than double-normal level) can serve as an immunological criterion for predicting viremia and leukopenia. It is also characteristic of a weak immune response.

## Conclusion

Increasing levels of IFN-α, IFN-γ, ICL, CD8, CD16, and CD20 (higher than double-normal values) can predict an adequate immune response to infection in patients with varicella.

Conversely, an insufficient increase (less than double-normal values) in IL-6, IFN-γ, ICL, CD16, and CD20 is predictive of severe and complicated forms of chickenpox. In these cases, the immune response to varicella would be weak and may require immunocorrective therapy.
